# Supplementary material for: Dual Disorders in the Consultation Liaison Addiction Service: Gender Perspective and Quality of Life
Source: J Clin Med. 2021 Nov 26;10(23):5572. doi: 10.3390/jcm10235572 (PMC8658653; doi:10.3390/jcm10235572)
Supplement: Supplementary file 1 [file jcm-10-05572-s001.zip › 2021_12_02_1442332 supplementary_rev.pdf]

Supplementary material

	Participants (n=68)	Lockdown (n=77)	
	**n%**	**n%**	**p**
**Gender:** Women	13 (19.1)	14 (18.1)	0.885
**Age ($\bar{x}$±SD) years**	50.99±11.67	50.1±11.7	0.647
**Civil status**			0.783
Single	28 (41.1)	34 (48)	
Married/partner	16 (23.5)	20 (25.9)	
Others	24 (35.2)	23 (29.8)	
**Origin**			0.128
National	48 (70.6)	20 (25.9)	
**Employment situation**			0.778
Employed	8 (11.7)	11 (14.2)	
Unemployed	33 (48.5)	32 (41.5)	
Retired	15 (22.1)	15 (19.4)	
Others	12 (17.6)	19 (24.6)	
**Living with**			**0.049**
Alone	13 (19.7)	14 (18.2)	
Family	30 (45.5)	24 (31.2)	
Homeless	16 (24.2)	14 (27.3)	
Others	9 (13.2)	25 (32.4)	
**Criminal records**			0.786
No	43 (63.2)	47 (61)	

Table S1. Sociodemographic characteristics of participant patients vs lockdown period patients.

SD: standard deviation；Bold numbers represent statistically significant results.

Table S2. Clinical characteristics of participant patients vs lockdown period patients.

	Participants (n=68)	Lockdown (n=77)	
	**n(%)**	**n(%)**	**p**
**Main drug:**			0.266
Opiates	26 (38.2)	23 (29.8)	
Alcohol	32 (47.1)	40 (51.9)	
Cocaine	7 (10.3)	4 (5.2)	
Amphetamines	2 (3)	1 (1.3)	
Tobacco	1 (1.5)	8 (10.3)	
Benzodiazepines	0	1 (1.3)	
**Starting age main drug ($\bar{x}$±SD) years**	18.71±6.59	19.17±9.5	0.747
**Total abstinence main drug ($\bar{x}$±SD) months**	24.15±35.27	19.86±46	0.555
**Previously linked to addiction treatment**	40 (58.9)	45 (58.4)	0.695
**Age at first addiction treatment ($\bar{x}$±SD) years**	34.74±14.29	36.03±11.8	0.693
**Time since last consumption of the main drug ($\bar{x}$±SD) months**	4.37±15.54	5.86±29.2	0.704
**Ab. HIV serology positive**	13 (19.1)	14 (18.8)	0.818

**Ab. HCV serology positive**	20 (29.4)	24 (31.1)	0.109
**Ab. core HBV serology positive**	14 (20.6)	14 (18.8)	0.065
**Ag. surface HBV positive**	2 (2.9)	3 (3.9)	0.091
**Chronic liver disease**	24 (35.3)	29 (37.6)	0.175

SD: Standard deviation, SUD: substance use disorder, HIV: human immunodeficiency virus, HBV: hepatitis B virus, HCV: hepatitis C virus, Ag. Antigen, Ab. Antibody.
